# Emotional Tone of Dreams and Daily Affect

**DOI:** 10.1093/geroni/igaa057.1379

**Published:** 2020-12-16

**Authors:** Cassandra Richardson, Taylor Vigoureux, Soomi Lee

**Affiliations:** University of South Florida, Tampa, Florida, United States

## Abstract

One theoretical function of dreams is emotion processing. However, few studies have examined how daily emotions in waking life (i.e., daytime affect) affect the emotional tone of dreams (i.e., dream affect) that night, and vice versa. This study examined daily bidirectional associations between dream affect and daytime positive and negative affect. Participants were 61 nurses who completed 2-weeks of ecological momentary assessments. If participants remembered the previous night’s dreams (nparticipants=50; ndays=268), they reported the dream’s emotional tone upon waking (‘0’=very negative to ‘100’=very positive). Participants also responded to a short-version of the Positive and Negative Affect Scale three times/day. Multilevel modeling was used to evaluate two temporal directions (dream affect→ daytime affect or daytime affect→ dream affect) at the within- and between-person levels. After adjusting for sociodemographic covariates, at the within-person level, daily positive affect was higher and daily negative affect was lower than usual on days following more positive dream affect (B=0.19, p<.05; B=-0.26, p<.05, respectively). When we added the other temporal direction, today’s positive or negative affect was not associated with dream affect that night. At the between-person level, nurses who reported more positive dream affect also reported more positive daytime affect (B=0.52, p<.01), but not less negative daytime affect (B=-0.34, p>.10). Findings suggest that dream affect is predictive of daily affect, but not the other way around. Future studies could further examine if emotions closer to sleep are more strongly associated with dream affect to motivate more precisely-timed affect interventions.

